# Prognosis of pediatric‐onset inflammatory bowel disease associated with primary sclerosing cholangitis: A population‐based study

**DOI:** 10.1002/jpn3.70176

**Published:** 2025-08-12

**Authors:** Marie‐Laura Godet, Hélène Sarter, Frédéric Gottrand, Colin Saoudi, Alexandre Louvet, Mathurin Fumery, Guillaume Savoye, Ariane Leroyer, Corinne Gower‐Rousseau, Delphine Ley, Madeleine Aumar

**Affiliations:** ^1^ Gastroenterology, Hepatology, and Nutrition Unit, Department of Pediatrics CHU Lille Lille France; ^2^ U1286 – INFINITE – Institute for Translational Research in Inflammation, Univ. Lille, Inserm, CHU Lille Lille France; ^3^ Public Health, Epidemiology and Economic Health Unit Epimad Registry, Maison Régionale de la Recherche Clinique, CHU Lille Lille France; ^4^ Division of Gastroenterology and Hepatology Univ. Lille, Inserm, CHU Lille, U1286 – INFINITE – Institute for Translational Research in Inflammation Lille France; ^5^ Gastroenterology Unit, Amiens University Hospital, and Peritox, UMRI01 Université de Picardie Jules Verne Amiens France; ^6^ Gastroenterology Unit, Rouen University Hospital, UMR 1073 University of Rouen Normandy Rouen France; ^7^ Research Unit and Public Health, Reims University Hospital Reims France

**Keywords:** cancer, children, Crohn's disease, liver disease, mortality, treatment, ulcerative colitis

## Abstract

**Objectives:**

To assess whether the prognosis of pediatric‐onset inflammatory bowel disease (IBD) is influenced by its association with primary sclerosing cholangitis (PSC) considering medical treatment, bowel resection, risk of cancer, and mortality.

**Methods:**

A retrospective population‐based study was conducted using data from the EPIMAD Registry, one of the most extensive prospective population‐based IBD studies globally. Cases (IBD‐PSC) were compared with matched controls (matched‐IBD). Inclusion criteria were age ≤17 years at IBD diagnosis and follow‐up ≥2 years. PSC was confirmed by magnetic resonance cholangiopancreatography and/or histology. Each case was matched to four controls by propensity score (i.e., sex, age, year, and location of IBD at diagnosis).

**Results:**

We included 24 cases and 96 controls. Median duration of follow‐up was 6.4 years (interquartile range = 3.1–14.3). No significant difference was observed between the two groups in terms of exposure to treatment at 5 years (immunosuppressants, corticosteroids, or antitumor necrosis factor). In IBD‐PSC, cancers were 28 times more frequent (standardized incidence ratio = 27.9; 95% confidence interval [CI], 7.0–111.7, *p* = 0.002), and death was 13 times more frequent (standardized mortality ratio = 13.3; 95% CI, 3.3–53.4, *p* = 0.010) than in the general population. No increased risk of cancer or mortality was observed in patients with IBD but without PSC compared to the general population.

**Conclusion:**

Although the course of IBD is not different, the prognosis of pediatric‐onset IBD associated with PSC is significantly worse than that of pediatric‐onset IBD without PSC because of increased cancer and mortality rates.

## Abstract (highlights)

**What is Known:**

- Primary sclerosing cholangitis (PSC) is a rare and severe disease in pediatrics.

**What is New:**

- Risk of cancer and death in young adulthood is increased in pediatric‐onset inflammatory bowel disease (IBD) associated with PSC compared with IBD alone.
- This study supports patient care by developing close follow‐up to detect liver complications and early complications, such as colonic or liver cancers.

## 1 INTRODUCTION

Inflammatory bowel disease (IBD) is associated with 76% of cases of primary sclerosing cholangitis (PSC), a rare chronic liver disease (0.2 cases per 100,000 children in the United States).^1^, ^2^ Its pathophysiology appears to be multifactorial, involving genetic predisposition, environmental factors such as alterations in gut microbiota, and changes in immune system reactions.^2^, ^3^, ^4^, ^5^ PSC means that no causal pathology has been individualized to explain sclerosing cholangitis. In addition, autoimmune sclerosing cholangitis is not always easily distinguished from autoimmune hepatitis (AIH) with overlap. Two recent studies hypothesized that autoimmune sclerosing cholangitis and AIH represent different inflammatory phases of PSC.^6^, ^7^ The prognosis of PSC is poor, leading to the progressive development of secondary biliary cirrhosis, whose complications (i.e., portal hypertension and/or hepatocellular insufficiency) can be life‐threatening and whose treatment can require liver transplantation.^1^, ^8^, ^9^ Rare cases of cholangiocarcinoma during childhood (median age, 15–18 years) were also described.^8^ Several adult series have shown that when associated with PSC, IBD seems to have a different phenotype compared with isolated IBD (e.g., lower activity score, less use of biologics, and fewer hospitalizations for IBD reasons) but a higher frequency of colorectal cancers.^4^, ^10^, ^11^, ^12^, ^13^, ^14^ Several pediatric series have confirmed a less severe phenotype of IBD when associated with PSC. However, to our knowledge, few studies to date have studied the cancer risk in this population, either for the medium or long term.^1^, ^8^, ^15^, ^16^, ^17^, ^18^

The aim of the present study was to compare the medium‐term severity of pediatric‐onset IBD, whether associated with PSC, in terms of mortality, cancer occurrence, intestinal resection, and exposure to treatments. We also aimed to compare the characteristics of IBD in our population associated with PSC with a reference population of IBD without PSC.

## 2 METHODS

### 2.1 Ethics statement

Use of the EPIMAD Registry was approved by the National Commission on Informatics and Liberties (CNIL No. 917089).

### 2.2 Study design

We conducted a retrospective, population‐based study. All patients from the EPIMAD Registry,^19^ diagnosed before the age of 17 with IBD between 1988 and 2019, associated with a radiologically and/or histologically proven PSC (including during adulthood), and a follow‐up of at least 2 years, were included in the cases group and identified as the “IBD‐PSC” group. We excluded patients with uncertain PSC (i.e., those without a biopsy or magnetic resonance cholangiopancreatography [MRCP] to confirm PSC).

Each patient with IBD‐PSC was matched to four controls extracted from the pediatric population in the EPIMAD Registry^20^ to obtain comparable groups at diagnosis, thereby reducing bias in the subsequent analyses of further evolution of the disease. This control group was referred to as the “matched‐IBD” group. Matching was performed using the propensity score method on the following variables: age, year of diagnosis, sex, and location of the disease at IBD diagnosis (according to the Paris Classification),^21^ with exact matching on sex, age ±3 years and disease location (for ulcerative colitis [UC], E3 and E4 are grouped to enable matching).

### 2.3 Data collection

The EPIMAD Registry is a population‐based IBD registry in northern France (comprising 9.3% of the entire French population) that prospectively collects all incident cases of IBD since 1988. The details of the methodology of this registry have been reported previously.^19^ IBD is defined by combining clinical, endoscopic, radiological, and biological criteria. Data were assessed in a blinded manner by two independent gastroenterologists. PSC was confirmed by histology on liver biopsy and/or endoscopic retrograde cholangiopancreatography and/or MRCP. Data were collected from the medical records of each patient at each visit, from diagnosis to the point of maximal follow‐up. These data were completed with hepatologic data retrospectively extracted from hospital medical records.

For all patients, we collected information on the age of the patient at IBD diagnosis, type and location of IBD (according to the Paris Classification),^21^ extraintestinal manifestations, treatments received, need for surgery, occurrence of cancers, and mortality. Surgery was restricted to intestinal resection, defined as partial or total colectomy, ileocecal resection, or small‐bowel resection.

### 2.4 Follow‐up

Patients had a follow‐up of at least 2 years, as documented in their medical files. To determine vital status, the identification (including name, surname, sex, date, and place of birth) of each patient in the cohort was cross‐linked with computerized French death certificates up to December 31, 2019. For the study of mortality, follow‐up was conducted from the date of IBD diagnosis until the end of 2019 for all patients.

Data on cancer diagnosis were obtained only from medical files during follow‐up. As a result, follow‐up is shorter for calculating cancer incidence than for mortality, resulting in different numbers of person‐years for cancer and mortality analyses.

### 2.5 Reference data

For cancer, reference data at the national level were obtained from the French Network of Population‐based Cancer Registries for the period 1990–2015.^22^ It was assumed that the cancer incidence for 1988 and 1989 was similar to that of 1990 and that the cancer incidence after 2016 was similar to that of 2015. Reference data for all‐cause mortality at the regional level were extracted from the National Institute of Statistics and Economic Studies (INSEE: https://www.insee.fr/). Death statistics and data by sex and 5‐year age groups in the area covered by EPIMAD for the period 1988–2015 were provided by the Regional Observatory for Health and Social of North France (Observatoire Régional de la Santé et du Social des Hauts‐de‐France). It was assumed that the death rate after 2016 was similar to that of 2015. For each of the four administrative areas, yearly population data by age group and sex were obtained from the INSEE census data.

### 2.6 Statistical analyses

Continuous variables are expressed as median and interquartile range (IQR), and categorical variables are expressed as frequency (percentage). Intergroup comparisons of continuous variables were performed using the Wilcoxon–Mann–Whitney test. Intergroup comparisons of qualitative variables were performed using chi‐squared or Fisher's exact tests (depending on the number of expected events).

Cumulative probabilities and 95% confidence intervals (CIs) for each treatment and surgery were estimated and described using the Kaplan–Meier method, and compared using a log‐rank test. The starting point was the IBD diagnosis.

The standardized mortality ratio (SMR) and standardized incidence ratio for cancer (SIR) were calculated. All deaths occurring until 2019 were considered. Only cancers that occurred during follow‐up were considered. Consequently, person‐years were calculated separately for mortality and cancer, which comprised invasive cancer of all sites, excluding cutaneous cancer except for melanoma. Expected cases of cancer and deaths were estimated by indirect standardization by sex and 5‐year age groups. Results were expressed as standardized incidence and mortality ratios (SIR and SMR, respectively) with 95% CIs estimated by an approximate method based on gamma distribution.^23^

Statistical analyses were performed using SAS software (version 9.4; SAS Institute) and R software (version 3.6.1; R Foundation for Statistical Computing). For SIR and SMR calculation, the epitools package was used.^24^ The threshold for statistical significance was set to *p* ≤ 0.05.

## 3 RESULTS

### 3.1 Patient characteristics

A total of 24 cases (IBD‐PSC) were included and matched with 96 controls (IBD). Diagnosis of PSC represents 1.2% of the entire pediatric population in the EPIMAD Registry. In both groups, 58% were boys (*n* = 14 and 56 in the IBD‐PSC and matched‐IBD groups, respectively). In both groups, 70% suffered from UC (17 cases in the IBD‐PSC group), and there was no IBD‐unclassified in our patients. The median age at diagnosis of IBD for both cases and controls was 13 years (IQR = 12–15 and 10–15, respectively). The median follow‐up was 7.0 and 6.3 years in the IBD‐PSC and matched‐IBD groups, respectively (IQR = 3.1–14.1 and 3.1–11.1, respectively) (Table 1). By construction (i.e., matching), the IBD‐PSC and matched‐IBD groups were comparable in terms of sex, age at diagnosis, and disease location. Extraintestinal manifestations were present in 28% (*n* = 4) of patients with IBD‐PSC versus 8% (*n* = 8) of patients with matched‐IBD (*p* = 0.255). In our population, the time interval between the diagnosis of IBD and PSC was 1.0 year (IQR = 0.2–3.2). For two patients, both diagnostics were concomitant.

**Table 1:** Comparison between IBD‐PSC, IBD, and total IBD pediatric cohort in terms of demographics and IBD phenotype at diagnosis.

	IBD‐PSC (*n* = 24)	Matched‐IBD (*n* = 96)	Total pediatric IBD population (*n* = 1344)	*p* (IBD‐PSC vs. IBD total population)
Sex (male), *n* (%)	14 (58.3%)	56 (58.1%)	700 (52.1%)	0.543
Age at diagnosis, median (IQR)	13.0 (12.0–14.5)	13.0 (10.5–15.0)	14.3 (11.7–16.0)	0.256
Follow‐up (years), median (IQR)	7.0 (3.1–14.1)	6.3 (3.1–11.1)	8.3 (4.3–13.9)	0.333
Type of IBD				<0.0001
CD	7 (29.2%)	28 (29.2%)	1007 (74.9%)	
UC	17 (70.8%)	68 (70.8%)	337 (25.1%)	
CD location^a^				0.518
L1	0	0	215 (21.6%)	
L2	2 (28.6%)	8 (28.6%)	234 (23.5%)	
L3	5 (71.4%)	20 (71.4%)	546 (54.9%)	
L4	0 (0.0%)	12 (12.5%)	296 (29.4%)	0.114
CD behavior^b^				1.00
B1	5 (83.3%)	22 (78.6%)	827 (82.4%)	
B2	1 (16.7%)	6 (21.4%)	145 (14.4%)	
B3	0 (0.0%)	0 (0.0%)	32 (3.2%)	
Extraintestinal manifestations	4 (28.6%)	8 (8.3%)	260 (25.9%)	0.080
Anoperineal lesions	1 (14.3%)	0 (0.0%)	41 (4.1%)	0.257
UC location^c^				0.008
E1	1 (5.9%)	4 (5.9%)	79 (23.7%)	
E2	2 (11.8%)	8 (11.8%)	107 (32.1%)	
E3	6 (35.3%)	15 (22.0%)	40 (12.0%)	
E4	8 (47.0%)	41 (60.3%)	107 (32.1%)	

Abbreviations: CD, Crohn's disease; IBD, inflammatory bowel disease; IBD‐PSC, inflammatory bowel disease associated with primary sclerosing cholangitis; IQR, interquartile range; UC, ulcerative colitis.

^a^ L1 = Distal 1/3 ileal + ‐limited cecal disease, L2 = colonic, L3 = Ileocolonic, L4 = Upper disease (L4a: upper disease proximal to Ligament of Treitz, L4b: Upper disease distal to Ligament of Treitz and proximal to distal 1/3 ileum).

^b^ B1 = Non‐stricturing non penetrating, B2 = Stricturing, B3 = Penetrating.

^c^ E1 = Ulcerative proctitis, E2 = Left side UC (distal to splenic flexure), E3 = Extensive (hepatic flexure distally), E4 = Pancolitis (proximal to hepatic flexure).

Two patients had small‐duct PSC. All patients underwent an MRCP, and every patient except four had a liver biopsy at diagnosis. Suspicion of PSC was raised by jaundice, cholestatic pruritus, and/or γ‐glutamyl transferase elevation. Four patients presented with PSC with features of autoimmunity (previously defined as PSC‐AIH overlap). Histological criteria for AIH were the presence of interface hepatitis, plasma cell predominance in the portal inflammatory infiltrate, regenerative rosettes, and emperipolesis. Five patients received liver transplantation due to multiple cholangitis and/or biliary cirrhosis.

### 3.2 Comparison of the IBD‐PSC group with the total pediatric IBD population

Patients with IBD‐PSC and those in the total pediatric IBD population were comparable in terms of sex, age at diagnosis, location, and duration of follow‐up (*p* = 0.54, 0.26, and 0.33, respectively). Compared with the total pediatric IBD population, patients with IBD‐PSC had more frequent UC than Crohn's disease (CD) (71% vs. 25%; *p* < 0.0001). In patients with IBD‐PSC and UC, the location of the disease was more frequently E3 and E4 (i.e., extensive colitis and pancolitis) than in the total pediatric IBD population (35% vs. 12% and 47% vs. 32%, respectively; *p* = 0.008). There was no difference in the location and behavior of CD, extraintestinal manifestations, or anoperineal lesions between the two populations (see Table 1).

### 3.3 Mortality

The mortality risk for patients with IBD‐PSC (SMR) was 13.3 times higher than in the general population (95% CI, 3.3–53.4) (see Table 2). In the IBD‐PSC population, the two deaths during young adulthood (28 and 29 years) were due to a liver complication: a cholangiocarcinoma and a septic shock in the context of liver failure in two non‐grafted patients. No mortality was observed in the IBD group. The death rate was 645/10^5^ person‐years compared to 80/10^5^ in the total pediatric IBD population^20^ (*p* < 0.0001).

**Table 2:** SMR and SIR of cancer in the IBD‐PSC population (*n* = 24) and IBD‐only population (*n* = 96).

		Person‐years	Observed cases	Expected numbers	SIR and 95% CI	*p*
Deaths	IBD‐PSC	310	2	0.150	13.3 (3.3–53.4)	0.010
	Matched‐IBD	1564	0	0.764	–	–
Cancers	IBD‐PSC	229	2	0.072	27.9 (7.0–111.7)	0.002
	Matched‐IBD	883	0	0.254	–	–

Abbreviations: 95% CI, 95% confidence interval; CD, Crohn's disease; IBD, inflammatory bowel disease; IBD‐PSC, inflammatory bowel disease associated with primary sclerosing cholangitis; SIR, standardized incidence ratio; SMR, standardized mortality ratio.

### 3.4 Cancer

The cancer risk for patients with IBD‐PSC (SIR) was 27.9 times higher than in the general population (95% CI, 7–111.7) (see Table 2). In the IBD‐PSC population, the two cancers were one colonic and one cholangiocarcinoma. No cancer was observed in the IBD group. Cancer incidence was 873/10^5^ person‐years compared to 108/10^5^ in the total pediatric IBD population^20^ (*p* < 0.0001).

### 3.5 Medical therapy

The cumulative probabilities from Kaplan–Meier analysis at 1 and 5 years for exposure to one of the three treatments of interest (i.e., immunosuppressants, corticosteroids, or anti‐TNF) were not different between IBD‐PSC and matched‐IBD groups.

**Figure 1:**
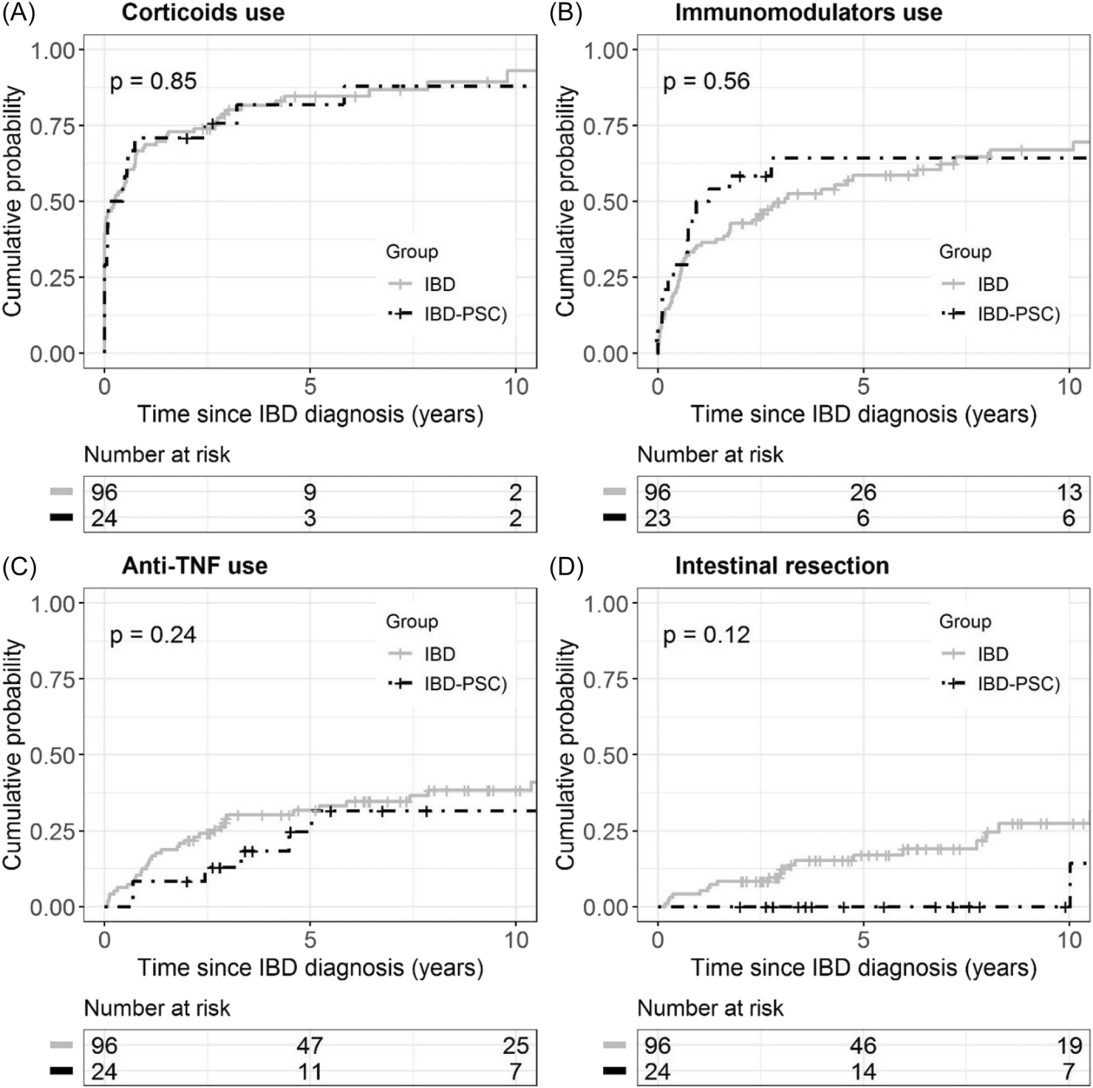
Comparison of treatment exposure and surgery between IBD‐PSC (black) and IBD (gray). (A–D) No statistical difference in the use of corticosteroids, immunosuppressants, anti‐TNF, and intestinal resection, respectively, between the two groups. IBD, inflammatory bowel disease; IBD‐PSC, inflammatory bowel disease with primary sclerosing cholangitis; TNF, tumor necrosis factor.

Concerning the use of systemic corticosteroids, the cumulative probabilities at 1 and 5 years were not different between IBD‐PSC and matched‐IBD groups (70.8%, 95% CI, 52.4–87.0 and 67.7%, 95% CI, 58.2–76.9 at 1 year; 81.8%, 95% CI, 63.0–94.6 and 84.1%, 95% CI, 75.3–91.0 at 5 years, respectively) (see Figure 1A).

Concerning the use of immunosuppressants, the cumulative probabilities at 1 and 5 years were not different between IBD‐PSC and matched‐IBD groups (47.8%, 95% CI, 30.0–69.5 and 35.4%, 95% CI, 26.8–45.8 at 1 year; 62.7%, 95% CI, 430–82.3 and 58.6%, 95% CI, 48.3–69.2 at 5 years, respectively) (see Figure 1B).

Concerning the use of anti‐tumor necrosis factor (TNF), the cumulative probabilities at 1 and 5 years were not different between IBD‐PSC and matched‐IBD groups (8.3%, 95% CI, 2.1–29.4 and 12.5%, 95% CI, 7.3–21.0 at 1 year; 24.6%, 95% CI, 10.8–50.2 and 31.7%, 95% CI, 23.1–42.4 at 5 years, respectively) (see Figure 1C).

### 3.6 Intestinal resection

The cumulative rate of surgery at 5 years was not statistically different between IBD‐PSC and matched‐IBD groups (0% and 17.0%, 95% CI, 10.3–27.4, respectively) (see Figure 1D).

## 4 DISCUSSION

Our study shows that the association of PSC with pediatric‐onset IBD significantly worsens the prognosis, increasing the mortality rate and the cancer risk in a young population compared with IBD alone. In the IBD‐PSC group, cancers and death were 28 and 13 times, respectively, more frequent than in the general population and higher than in the total IBD population. However, these results are to be interpreted with caution, given the wide CI. The excess morbidity and mortality risk was previously described in adult cohorts, mainly due to portal hypertension, hepatic failure, cholangiocarcinoma, or colorectal cancer.^5^, ^11^, ^12^, ^25^, ^26^ In our cohort, deaths were related to PSC (e.g., decompensated liver failure and cholangiocarcinoma).

The description of our study population is similar to that in the literature: IBD‐PSC was more frequent in male patients; PSC was associated with UC in 72%,^2^, ^17^, ^26^, ^27^ whereas CD was more frequent in the entire pediatric IBD population. In our study, the two deaths were related to liver complications (e.g., cholangiocarcinoma and liver failure) at the ages of 28 and 29 years. Deneau et al. showed that global survival in children with PSC (with or without IBD) was significantly shorter than in the general population; in that cohort, liver transplantation occurred in 14% of patients with PSC at a median age of 15 years (IQR = 11.9–17.7). The overall event‐free survival rates at 5 and 10 years were 70% and 53%, respectively.^8^ In our study, 5 out of 24 patients with IBD‐PSC (20%) were transplanted during a median follow‐up of 7 years. In a multinational study of pediatric patients with IBD with a median follow‐up of 5 years, de Ridder et al. showed that the first cause of mortality was infection before cancer, with a median age at the time of death of 14.6 years.^28^

Another noteworthy finding of our study is the higher risk of cancer in patients with IBD‐PSC compared with the IBD‐control group. Both of our patients were young adults at the time of cancer diagnosis: 29 years for the cholangiocarcinoma (17 years after IBD and PSC diagnosis) and 30 years for the colorectal carcinoma (15 years after IBD diagnosis and 2 years before the diagnosis of PSC). Our results confirmed those of two pediatric multicenter studies.^27^, ^28^ Other studies have previously reported that the association of IBD with PSC in adults was a significant risk factor for colorectal cancer and cholangiocarcinoma.^5^, ^10^, ^11^, ^12^, ^14^ In an international pediatric study by de Ridder et al., hematopoietic tumors were the most frequent malignancies in all patients with IBD (45%), and the median disease duration before cancer diagnosis was 5 years.^28^ Another European pediatric international cohort published by Joosse et al. with a median follow‐up of 3 years reported malignancy as the first cause of death in patients with IBD; however, Joosse et al. did not find any significant difference in cancer incidence compared with the general population in the same age group.^29^ In the Pediatric PSC Consortium, El‐Matary et al. reported an incidence of colorectal dysplasia or cancer in pediatric PSC‐UC/IBD‐unclassified of 2.8 cases per 1000 person‐years.^30^ A recent meta‐analysis revealed a 2.4‐fold increase in the relative rate of all types of cancers among patients with pediatric‐onset IBD compared with the general pediatric population (total follow‐up time ranged from 8254 person‐years to 148,682 person‐years). This increased rate was mainly due to gastrointestinal cancers, with a low overall incidence risk (1.0–3.3 cases per 1,000 person‐years).^31^ This increased rate was not observed in our control group (i.e., IBD), likely due to the small sample size. Indeed, in the pediatric IBD population, the standardized ratio for cancer risk was 2.7 (95% CI, 1.5–4.8), which confirms the significantly increased risk in the IBD‐PSC group (*p* < 0.0001).^20^

Our study highlights the difference in UC phenotype previously described in the adult and pediatric literature when associated with PSC. Pancolitis or extensive colitis is more frequent in UC with PSC than in UC alone.^11^, ^12^, ^13^, ^15^, ^17^, ^18^, ^26^, ^27^, ^32^, ^33^ Several adult series suggest a less severe course of IBD when associated with PSC, with lower disease activity during flares, less use of biological therapy, and mild inflammation at biopsies.^10^, ^13^ We did not find any significant difference in the severity of IBD in terms of the use of immunosuppressants, corticosteroids, or anti‐TNF. Less use of immunosuppressive drugs in the IBD‐PSC group was reported in two previous pediatric studies.^16^, ^18^ In terms of intestinal resection, the difference found in our cohort (0% in the IBD‐PSC group vs. 17% in the matched‐IBD group) did not reach statistical significance (*p* = 0.12), as in the series by Lascurain et al.^15^ This might be due to a lack of statistical power in our cohort, due to the small numbers of patients. In adult cohorts, some authors did not find any statistical difference in terms of colectomy between the PSC‐IBD and IBD groups, whereas others found a higher rate of colectomy in the IBD‐PSC group. Reasons for colectomy were different between both groups: that is, therapy‐refractory colitis for patients with IBD or the development of neoplasia in the IBD‐PSC group.^11^

A limitation of our study is the relatively small size of our cohort, which hampers the analysis of some variables of interest due to a lack of statistical power, resulting in no significant results for the analysis of exposure to treatments and extremely wide CIs for SMR and SIR. PSC in children is a rare disease, even more so when associated with IBD. If the disease activity index was not recorded retrospectively, exposure to treatment, disease location, and phenotype were collected at each follow‐up visit, reflecting disease severity.

Despite the small sample size of this cohort, we obtained statistically significant results concerning mortality and cancer risk, yet with wide CIs. A major strength of our study is the use of data extracted from a large population‐based registry, characterized by a robust methodology and a long inclusion period. To our knowledge, the EPIMAD Registry is one of the world's largest IBD population‐based studies. Its strong methodology (i.e., standardized questionnaire, data collection by specialized investigators, and expert validation blinded to diagnoses) allows the exploitation of reliable and representative data in a large population area of our country (6 million inhabitants, 9.3% of the entire French population).^19^, ^32^ Its large nested pediatric cohort, including all cases of IBD diagnosed before the age of 17 years between 1988 and 2011 in the same area, benefited from the same strong methodology. Another strength of our study is its population‐based case–control study design, which is representative of a whole population basin within a French area rather than being selective from a single tertiary hospital. Moreover, the pairing between the IBD‐PSC and IBD groups strengthens the quality of our data and eliminates possible bias due to the size of the inclusion period, during which follow‐up and treatments for IBD underwent considerable changes.

## 5 CONCLUSIONS

The association of PSC with IBD severely worsens the prognosis in terms of death and cancer incidence. Close follow‐up to ensure early detection of both colonic and hepatic cancers seems necessary, particularly during adolescence and the transition to adulthood.

## CONFLICT OF INTEREST STATEMENT

The authors declare no conflicts of interest.

## References

[1] Deneau M , Jensen KM , Holmen J , Williams MS , Book LS , Guthery SL . Primary sclerosing cholangitis, autoimmune hepatitis, and overlap in Utah children: epidemiology and natural history. Hepatology. 2013;58(4):1392‐1400. 23686586 10.1002/hep.26454

[2] Adike A , Carey EJ , Lindor KD . Primary sclerosing cholangitis in children versus adults: lessons for the clinic. Expert Rev Gastroenterol Hepatol. 2018;12(10):1025‐1032. 30199272 10.1080/17474124.2018.1521719

[3] Del Chierico F , Cardile S , Baldelli V , et al. Characterization of the gut microbiota and mycobiota in Italian pediatric patients with primary sclerosing cholangitis and ulcerative colitis. Inflamm Bowel Dis. 2024;30(4):529‐537. 37696680 10.1093/ibd/izad203 PMC10988104

[4] Guerra I , Bujanda L , Castro J , et al. Clinical characteristics, associated malignancies and management of primary sclerosing cholangitis in inflammatory bowel disease patients: a multicentre retrospective cohort study. J Crohns Colitis. 2019;13(12):1492‐1500. 31063540 10.1093/ecco-jcc/jjz094

[5] Karlsen TH , Folseraas T , Thorburn D , Vesterhus M. Primary sclerosing cholangitis—a comprehensive review. J Hepatol. 2017;67:1298‐1323. 28802875 10.1016/j.jhep.2017.07.022

[6] Ricciuto A , Kamath BM , Hirschfield GM , Trivedi PJ . Primary sclerosing cholangitis and overlap features of autoimmune hepatitis: a coming of age or an age‐ist problem? J Hepatol. 2023;79(2):567‐575. 36870613 10.1016/j.jhep.2023.02.030

[7] Kellermayer R , Carbone M , Horvath TD , et al. Identifying a therapeutic window of opportunity for people living with primary sclerosing cholangitis: embryology and the overlap of inflammatory bowel disease with immune‐mediated liver injury. Hepatology. Published online May 14, 2024. 10.1097/HEP.0000000000000926 38743006

[8] Deneau MR , El‐Matary W , Valentino PL , et al. The natural history of primary sclerosing cholangitis in 781 children: a multicenter, international collaboration. Hepatology. 2017;66(2):518‐527. 28390159 10.1002/hep.29204

[9] Fagundes EDT , Ferreira AR , Hosken CC , Queiroz TCN . Primary sclerosing cholangitis in children and adolescents. Arq Gastroenterol. 2017;54:286‐291. 28977113 10.1590/S0004-2803.201700000-50

[10] Cordes F , Laumeyer T , Gerß J , et al. Distinct disease phenotype of ulcerative colitis in patients with coincident primary sclerosing cholangitis: evidence from a large retrospective study with matched cohorts. Dis Colon Rectum. 2019;62(12):1494‐1504. 31725582 10.1097/DCR.0000000000001496

[11] Sørensen JØ , Nielsen OH , Andersson M , et al. Inflammatory bowel disease with primary sclerosing cholangitis: a Danish population‐based cohort study 1977‐2011. Liver Int. 2018;38(3):532‐541. 28796371 10.1111/liv.13548

[12] Fraga M , Fournier N , Safroneeva E , et al. Primary sclerosing cholangitis in the Swiss inflammatory bowel disease cohort study: prevalence, risk factors, and long‐term follow‐up. Eur J Gastroenterol Hepatol. 2017;29(1):91‐97. 27622999 10.1097/MEG.0000000000000747

[13] Palmela C , Peerani F , Castaneda D , Torres J , Itzkowitz SH . Inflammatory bowel disease and primary sclerosing cholangitis: a review of the phenotype and associated specific features. Gut Liver. 2018;12(1):17‐29. 28376583 10.5009/gnl16510 PMC5753680

[14] Shah SC , Ten Hove JR , Castaneda D , et al. High risk of advanced colorectal neoplasia in patients with primary sclerosing cholangitis associated with inflammatory bowel disease. Clin Gastroenterol Hepatol. 2018;16(7):1106‐1113.e3. 29378311 10.1016/j.cgh.2018.01.023

[15] Lascurain L , Jensen MK , Guthery SL , Holmen J , Deneau M . Inflammatory bowel disease phenotype in pediatric primary sclerosing cholangitis. Inflamm Bowel Dis. 2016;22(1):146‐150. 26619054 10.1097/MIB.0000000000000586 PMC6459684

[16] Ricciuto A , Hansen BE , Ngo B , et al. Primary sclerosing cholangitis in children with inflammatory bowel diseases is associated with milder clinical activity but more frequent subclinical inflammation and growth impairment. Clin Gastroenterol Hepatol. 2020;18(7):1509‐1517.e7. 31493578 10.1016/j.cgh.2019.08.048

[17] Bramuzzo M , Martelossi S , Torre G , et al. Clinical features and risk factors of autoimmune liver involvement in pediatric inflammatory bowel disease. J Pediatr Gastroenterol Nutr. 2016;63(2):259‐264. 26756875 10.1097/MPG.0000000000001078

[18] Shiau H , Ihekweazu FD , Amin M , Fofanova T , Miloh T , Kellermayer R . Unique inflammatory bowel disease phenotype of pediatric primary sclerosing cholangitis: a single‐center study. J Pediatr Gastroenterol Nutr. 2017;65(4):404‐409. 28141677 10.1097/MPG.0000000000001531 PMC5533626

[19] Gower‐Rousseau C , Salomez JL , Dupas JL , et al. Incidence of inflammatory bowel disease in Northern France (1988‐1990). Gut. 1994;35(10):1433‐1438. 7959201 10.1136/gut.35.10.1433 PMC1375020

[20] Dupont‐Lucas C , Leroyer A , Ley D , et al. Increased risk of cancer and mortality in a large French population‐based paediatric‐onset inflammatory bowel disease retrospective cohort. J Crohns Colitis. 2023;17(4):524‐534. 36316987 10.1093/ecco-jcc/jjac166

[21] Levine A , Griffiths A , Markowitz J , et al. Pediatric modification of the Montreal classification for inflammatory bowel disease: the Paris classification. Inflamm Bowel Dis. 2011;17(6):1314‐1321. 21560194 10.1002/ibd.21493

[22] Bossard N , Velten M , Remontet L , et al. Survival of cancer patients in France: a population‐based study from the association of the French Cancer Registries (FRANCIM). Eur J Cancer. 2007;43(1):149‐160. 17084622 10.1016/j.ejca.2006.07.021

[23] Fay MP , Kim S . Confidence intervals for directly standardized rates using mid‐p gamma intervals. Biometrical J. 2017;59(2):377‐387. 10.1002/bimj.201600111 28008645

[24] Aragon TJ , Fay MP . Epitools: Epidemiology Tools. 2020.

[25] Loftus EV . PSC‐IBD: a unique form of inflammatory bowel disease associated with primary sclerosing cholangitis. Gut. 2005;54(1):91‐96. 15591511 10.1136/gut.2004.046615 PMC1774346

[26] Lee WS , Karthik SV , Ng RT , et al. Characteristics and outcome of primary sclerosing cholangitis associated with inflammatory bowel disease in Asian children. Pediatr Neonatol. 2019;60(4):396‐404. 31409456 10.1016/j.pedneo.2018.09.007

[27] Boonstra K , van Erpecum KJ , van Nieuwkerk KMJ , et al. Primary sclerosing cholangitis is associated with a distinct phenotype of inflammatory bowel disease. Inflamm Bowel Dis. 2012;18(12):2270‐2276. 22407885 10.1002/ibd.22938

[28] de Ridder L , Turner D , Wilson DC , et al. Malignancy and mortality in pediatric patients with inflammatory bowel disease: a multinational study from the Porto Pediatric IBD Group. Inflamm Bowel Dis. 2014;20(2):291‐300. 24374875 10.1097/01.MIB.0000439066.69340.3c

[29] Joosse ME , Aardoom MA , Kemos P , et al. Malignancy and mortality in paediatric‐onset inflammatory bowel disease: a 3‐year prospective, multinational study from the paediatric IBD porto group of ESPGHAN. Aliment Pharmacol Ther. 2018;48(5):523‐537. 29984520 10.1111/apt.14893

[30] El‐Matary W , Guthery SL , Amir AZ , et al. Colorectal dysplasia and cancer in pediatric‐onset ulcerative colitis associated with primary sclerosing cholangitis. Clin Gastroenterol Hepatol. 2021;19(5):1067‐1070.e2. 32360820 10.1016/j.cgh.2020.04.055 PMC8788582

[31] Elmahdi R , Lemser CE , Thomsen SB , Allin KH , Agrawal M , Jess T . Development of cancer among patients with pediatric‐onset inflammatory bowel disease: a meta‐analysis of population‐based studies. JAMA Netw Open. 2022;5(3):e220595. 35230438 10.1001/jamanetworkopen.2022.0595 PMC8889462

[32] Ghione S , Sarter H , Fumery M , et al. Dramatic increase in incidence of ulcerative colitis and Crohn's disease (1988–2011): a population‐based study of French adolescents. Am J Gastroenterol. 2018;113(2):265‐272. 28809388 10.1038/ajg.2017.228

[33] Ricciuto A , Fish J , Carman N , et al. Symptoms do not correlate with findings from colonoscopy in children with inflammatory bowel disease and primary sclerosing cholangitis. Clin Gastroenterol Hepatol. 2018;16(7):1098‐1105.e1. 29378308 10.1016/j.cgh.2018.01.020

